# ﻿*Paraphlomis leigongshanensis* (Lamiaceae), a new species from Guizhou, China

**DOI:** 10.3897/phytokeys.267.157814

**Published:** 2025-12-02

**Authors:** Wei-Hao Yao, Guo-Bin Jiang, De-Hui Yu, Sheng Chen, Hong-Mei Chen, Li Dai, Bai-Qiu He, He Li

**Affiliations:** 1 Key Laboratory of National Forestry and Grassland Administration on Biodiversity Conservation in Karst Mountainous Areas of Southwestern China, Guizhou Academy of Forestry, Guiyang, 550005, China Key Laboratory of National Forestry and Grassland Administration on Biodiversity Conservation in Karst Mountainous Areas of Southwestern China, Guizhou Academy of Forestry Guiyang China; 2 Guizhou Leigong Mountain National Nature Reserve Administration, Leishan, 557100, China Guizhou Leigong Mountain National Nature Reserve Administration Leishan China; 3 Institute of Biology, Guizhou Academy of Sciences, Guiyang, 550009, China Institute of Biology, Guizhou Academy of Sciences Guiyang China; 4 College of Life Sciences, University of Chinese Academy of Sciences, Beijing, 100049, China University of Chinese Academy of Sciences Beijing China

**Keywords:** Guizhou, Lamioideae, new taxon, phylogenetic analyses

## Abstract

*Paraphlomis leigongshanensis* G.B. Jiang & W.H. Yao (Lamiaceae), a new species endemic to Leigong Mountain National Nature Reserve (Guizhou, China), is described and illustrated. Morphologically, it is most similar to *P. jiangyongensis*, but can easy be distinguished by its lamina shape, calyx teeth shape, and corolla color. A close relationship between the new species and P. gracilis var. lutienensis was revealed by molecular phylogenetic analyses based on ETS and ITS sequences, but they are morphologically distinct from each other. With only three known populations (ca. 200 mature individuals) in subtropical evergreen forests, the new species is assessed as Endangered (EN) under IUCN criteria B2ab(iii); D.

## ﻿Introduction

*Paraphlomis* (Prain) Prain (Lamiaceae, Lamioideae) is a member of the tribe Paraphlomideae Bendiksby, which was established to accommodate *Paraphlomis*, *Matsumurella* Makino, and *Ajugoides* Makino based on molecular phylogenetic evidence (Bendiksby et al. 2011; Zhao et al. 2021). Most species of *Paraphlomis* are distributed in southern China, with several taxa extending into the Himalayas, Korea, and Southeast Asia (Wu and Li 1977; Li and Hedge 1994a; Ko et al. 2014; Chen et al. 2021). Previous molecular phylogenetic studies revealed that *Paraphlomis* was not monophyletic, as species of *Matsumurella* were nested within it (Chen et al. 2021, 2022a; Guo et al. 2023). Recently, Yuan et al. (2024) treated *Matsumurella* and *Ajugoides* as synonyms of *Paraphlomis* based on plastome data and nuclear ribosomal DNA (nrDNA) sequences, as well as morphological evidence. Morphologically, *Paraphlomis* is characterized by its rhizomatous or stoloniferous habit, plants with simple hairs, nutlets with a truncate apex, and actinomorphic calyces (Wu and Li 1977; Bendiksby et al. 2011; Chen et al. 2021; Yuan et al. 2024). According to the Catalogue of Life China 2025 Annual Checklist and recent references, *Paraphlomis* comprises 51 species and six varieties (Li and Hedge 1994a; Chen et al. 2021; Yuan et al. 2024, 2025; Liu et al. 2025).

During a botanical field survey in Leigong Mountain National Nature Reserve, Guizhou Province, in 2024, we discovered an unknown species of *Paraphlomis*. This species is distinguished by its dwarf habit, a white corolla with pink stripes, and hairy mericarps. After careful field observations, morphological comparisons with other members of *Paraphlomis*, and molecular phylogenetic analyses, we confirmed its specific status and taxonomic placement within the genus. We hereby named it as *P. leigongshanensis* G.B. Jiang & W.H. Yao.

## ﻿Materials and methods

### ﻿Morphological study

During February 2024 and July 2025, four field surveys were carried out at the type locality of the new species. Specimens from several herbaria were examined, including GYBG, GZTM, HGAS, HIB, IBK, IBSC, KUN, NAS, and PE (herbarium abbreviations follow the conventions outlined by Thiers (2025)). Additionally, we consulted several online databases, including the China Plant Photography Database (http://ppbc.iplant.cn/), the Chinese Field Herbarium (http://www.cfh.ac.cn/), and Global Plants (http://plants.jstor.org/), to obtain supplementary information on species distributions and morphological data. To distinguish the new species from other species of *Paraphlomis*, we conducted detailed morphological comparisons, focusing on key characteristics such as stems, leaves, calyx, corolla, mericarps, and trichomes. All measurements were made using a dissecting microscope. The morphological description followed relevant taxonomic and floristic literature on *Paraphlomis*, with terminology standardized according to Li and Hedge (1994b), ensuring consistent and accurate morphological interpretation.

### ﻿Molecular phylogenetic analyses

For the molecular phylogenetic reconstruction of *Paraphlomis*, we included a total of 53 samples: 38 representing *Paraphlomis* taxa (34 species and four varieties) and two outgroup taxa. The outgroups comprised Phlomoides dentosa var. glabrescens (Danguy) C.L. Xiang & H. Peng and *Phlomis fruticosa* L. Only one accession of the new species was sampled and sequenced in this study, while sequence data for other species were obtained from GenBank and previous studies (Chen et al. 2021, 2022a, 2022b).

Genomic DNA was extracted from dried leaf tissue using a modified CTAB method (Doyle and Doyle 1987). Previous studies have shown that phylogenetic trees based on nuclear ribosomal DNA internal and external transcribed spacers (nrITS and nrETS) offer more effective insights into the phylogenetic relationships of *Paraphlomis* than those constructed using chloroplast DNA (Chen et al. 2021; Yuan et al. 2024). Thus, only the nrDNA data was used here for phylogenetic reconstruction. Voucher information and GenBank accession numbers for all sequences are provided in Appendix 1.

The best-fitting substitution model was selected using the Akaike Information Criterion (AIC) based on sequence data in jModelTest v.2.1.7 (Darriba et al. 2012). Phylogenetic analyses were performed using Bayesian Inference (BI) and Maximum Likelihood (ML) methods, employing MrBayes (Ronquist et al. 2012) and RAxML v.8.2.9 (Stamatakis 2014) on the CIPRES Science Gateway v.3.3 (Miller et al. 2010), respectively. The Markov chain Monte Carlo algorithm was run for 20 million generations with four incrementally heated chains starting from random trees and sampling one out of every 1,000 generations. The initial 25% of sampled trees were discarded as burn-in and the 50% majority-rule consensus tree was calculated from remaining trees with nodal support summarized as posterior probabilities (PP). All phylogenetic trees were visualized and edited for clarity using FigTree v.1.4.2 (Rambaut 2014), with PP and bootstrap support (BS) values annotated on the branches.

## ﻿Results and discussion

### ﻿Morphological comparison

**Figure A1.**
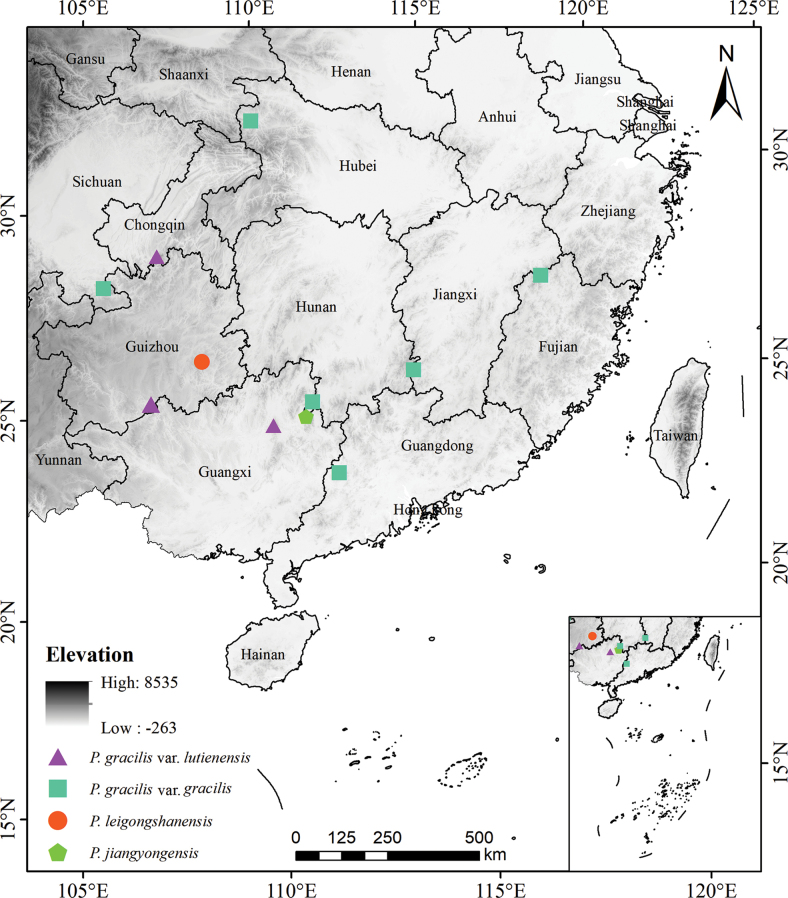
Distribution of *Paraphlomis leigongshanensis*, *P. jiangyongensis*, P. gracilis var. gracilis, and P. gracilis var. lutienensis.

Morphological comparisons were based on an examination of 196 specimens representing 14 species of *Paraphlomis*. A key diagnostic character of the potential new species *P. leigongshanensis* is the hairy mericarps, which serves as an important taxonomic feature for the infrageneric classification (Chen et al. 2021; Yuan et al. 2024). Morphologically, *P. leigongshanensis* is most similar to *P. jiangyongensis* in the dwarf habit ascending stems, and hairy mericarps (Table 1). However, it differs from *P. jiangyongensis* by its abaxially purple (vs. green) laminae, calyx teeth acuminate (vs. acute) at apex, and longer corolla tubes. Additionally, the corolla of *P. leigongshanensis* is white with pink stripes and spots, with the markings concentrated on the lower lip, differing from the pale yellow corolla lips with red spots in *P. jiangyongensis*. This species is distributed in the mid-subtropical evergreen broad-leaved forests in Leishan County, Guizhou, exhibiting geographical isolation from *P. jiangyongensis* (Appendix 2: Fig. A1).

**Table 1.** Morphological comparisons among *P. leigongshanensis*, *P. jiangyongensis*, P. gracilis var. gracilis, and P. gracilis var. lutienensis.

Character	*P. leigongshanensis*	*P. jiangyongensis*	P. gracilis var. gracilis	P. gracilis var. lutienensis
Stem	Prostrate to ascending, 5–10 cm tall	Ascending, 5–30 cm tall	Erect, 30–100 cm tall	Erect, 30–100 cm tall
Lamina	Ovate to long elliptic, 2.5–7 × 2–5 cm, abaxially purple, densely villose	Ovate to subcircular, 3–8 × 2–5 cm, abaxially light green, densely strigose	Lanceolate, 5–10 × 1.7–3.3 cm, abaxially green, densely strigose and glandular	Lanceolate, 6.5–10.5 × 1.7–2.5 cm, abaxially green, densely strigose and glandular, subsessile and narrowly lanceolate
Verticillaster	2–10-flowered	2–8-flowered	(2–) 4–8 (–12)-flowered	(2–) 4–8 (–12)-flowered
Pedicel	Sessile	Sessile	Subsessile	Ca. 4 mm long
Calyx	Purplish–red, densely villose	Green, densely strigose	Green, densely retrorse strigose	Green, densely retrorse strigose
Calyx teeth	4–8 mm long, triangular, apex acuminate	Ca. 2 mm long, triangular, apex acute	Ca. 6 mm long, triangular, apex acuminate or subulate	Ca. 4 mm long, triangular, apex acuminate
Corolla	12–17 mm long, white lower lip with pink stripes and spots	7.5–10 mm long, pale yellow, lower lip with red spots	15 mm long, upper yellow, white or yellowish green, lower lip white with purple spots or yellow with red spots	15 mm long, yellow, lower lip with pink spots

### ﻿Molecular phylogenetic analyses

The concatenated nrDNA dataset had a total length of 1259 bp, comprising 814 bp from the ITS region and 445 bp from the ETS region. The dataset contained 356 variable sites (168 from ITS, 188 from ETS) and 205 parsimony-informative characters (89 from ITS, 116 from ETS).

**Figure 1.**
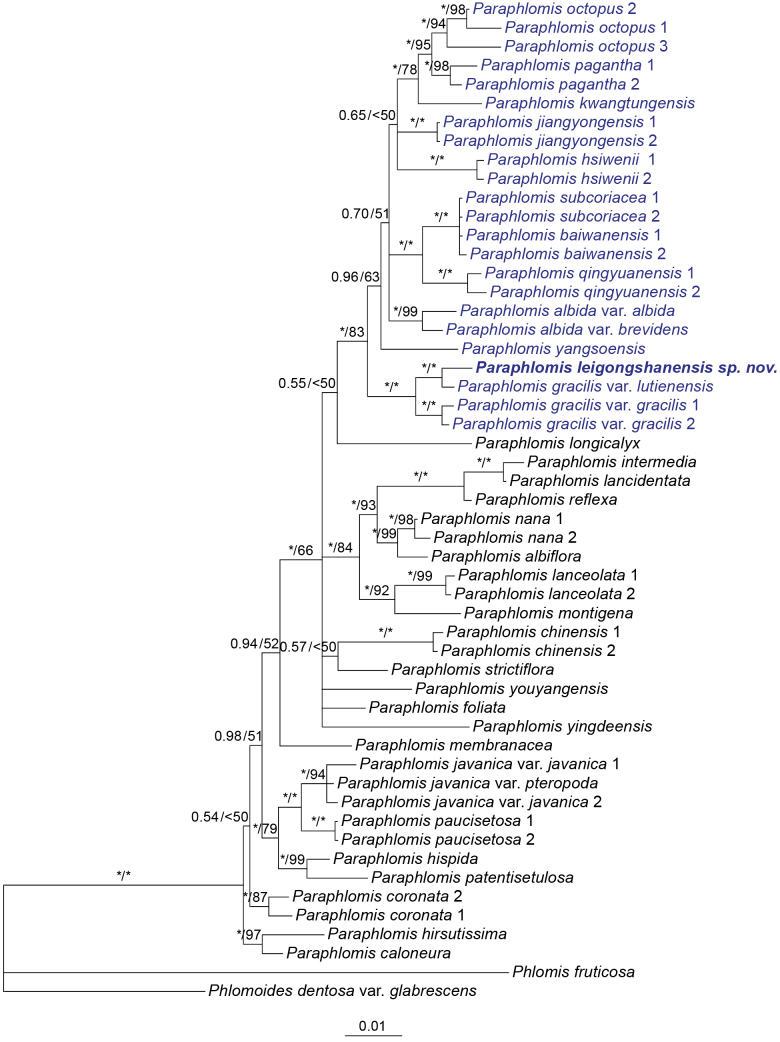
Bayesian 50% majority-rule consensus tree of *Paraphlomis* based on combined nuclear (ITS and ETS) data set. Support values ≥ 0.50 PP or 50% BS are displayed above the branches (an “*” indicates a support value of 1.00 PP or 100% BS). Multiple accessions of the same species are numbered according to Appendix 1

The resulting phylogenetic tree of *Paraphlomis* was consistent with previous studies (Chen et al. 2021; Yuan et al. 2024). The potential new species *P. leigongshanensis* was resolved as sister to P. gracilis var. lutienensis (Y. Z. Sun) C. Y. Wu (Fig. 1: BS = 100%/PP = 1.00), and the *P. leigongshanensis*–P. gracilis var. lutienensis clade was further sister to P. gracilis var. gracilis Kudô (Fig. 1: BS = 100% / PP = 1.00). The three taxa were all nested within the “Clade III” proposed by Chen et al. (2021) and Yuan et al. (2024) (Fig. 1: BS = 83%/PP = 1.00). Species in this clade (highlighted in blue in Fig. 1) are all characterized by hairy mericarps (Chen et al. 2021; Yuan et al. 2024). Despite the close relationships and geographical affinities (Appendix 2: Fig. A1) among *P. leigongshanensis*, P. gracilis var. gracilis, and P. gracilis var. lutienensis, *P. leigongshanensis* is morphologically distinct from the latter two taxa. Specifically, *P. leigongshanensis* exhibits a creeping habit with stems 5–10 cm tall, ovate to elliptic leaves, reddish-purple calyces, sessile flowers, and white corollas with pink stripes and spots. In contrast, P. gracilis var. gracilis has erect and much taller stems (up to 1 m tall), lanceolate leaves, green calyces, subsessile flowers, and yellowish green to white corollas with purple spots (Table 1). Paraphlomis gracilis var. lutienensis differs from both species by its subsessile and narrowly lanceolate laminae and yellow corollas. Moreover, a recent phylogenetic study based on plastome data (Yuan et al. 2024) indicated that the phylogenetic relationship between P. gracilis var. gracilis and P. gracilis var. lutienensis is consistent with the results of our study. Based on our phylogenetic results, although *P. leigongshanensis* is grouped with P. gracilis var. gracilis and P. gracilis var. lutienensis, there is sufficient evidence to support its recognition as a distinct species. The morphological differences further reinforce its taxonomic distinctiveness and provide additional support for its designation as a new species.

### ﻿Taxonomic treatment

#### ﻿ Paraphlomis leigongshanensis

Taxon classificationPlantaeLamialesLamiaceae

G.B.Jiang & W.H.Yao sp. nov.

03EC3675-9FC2-5141-9DF3-BCF248CF3EAE

urn:lsid:ipni.org:names:77365161-1

**Figure 2.**
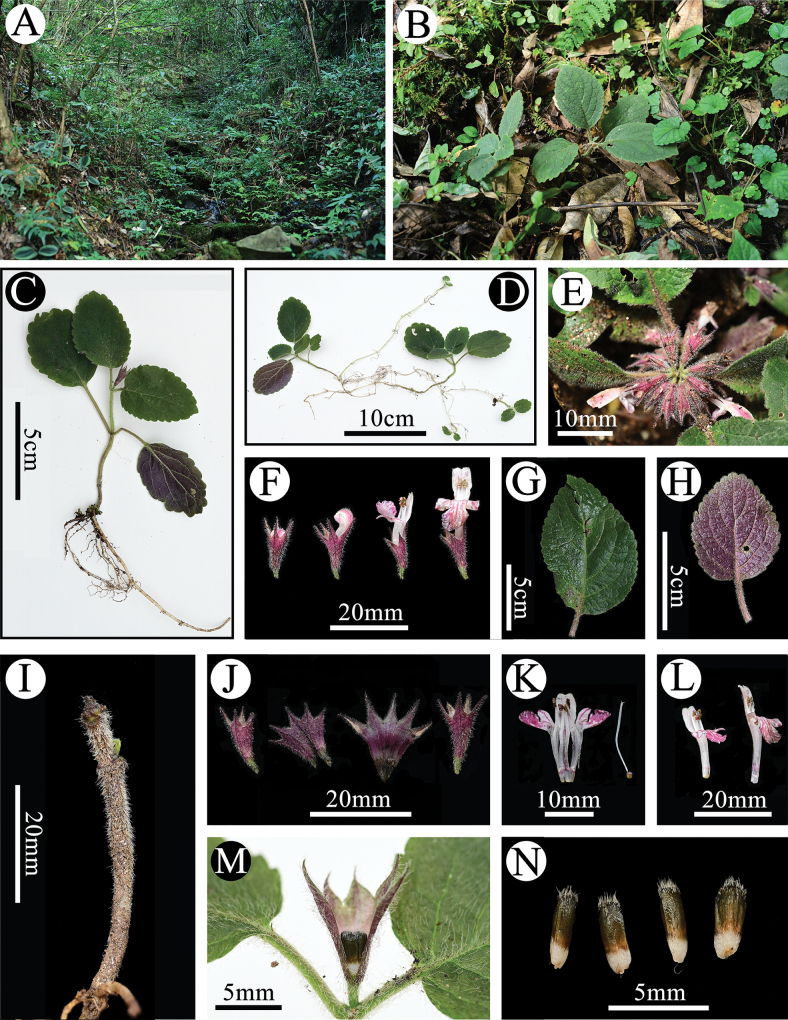
*Paraphlomis leigongshanensis* from the type locality. **A.** Habitat; **B-D.** Habit; **E.** Inflorescence; **F.** Flowering condition; **G.** Adaxial view of leaf; **H.** Abaxial view of leaf; **I.** Stem; **J.** Calyces; **K.** Dissected corolla and pistil; **L.** Corollas; **M.** Dissected calyx and mericarps; **N.** Dried mericarps. (Photographed by Guo-Bin Jiang).

**Figure 3.**
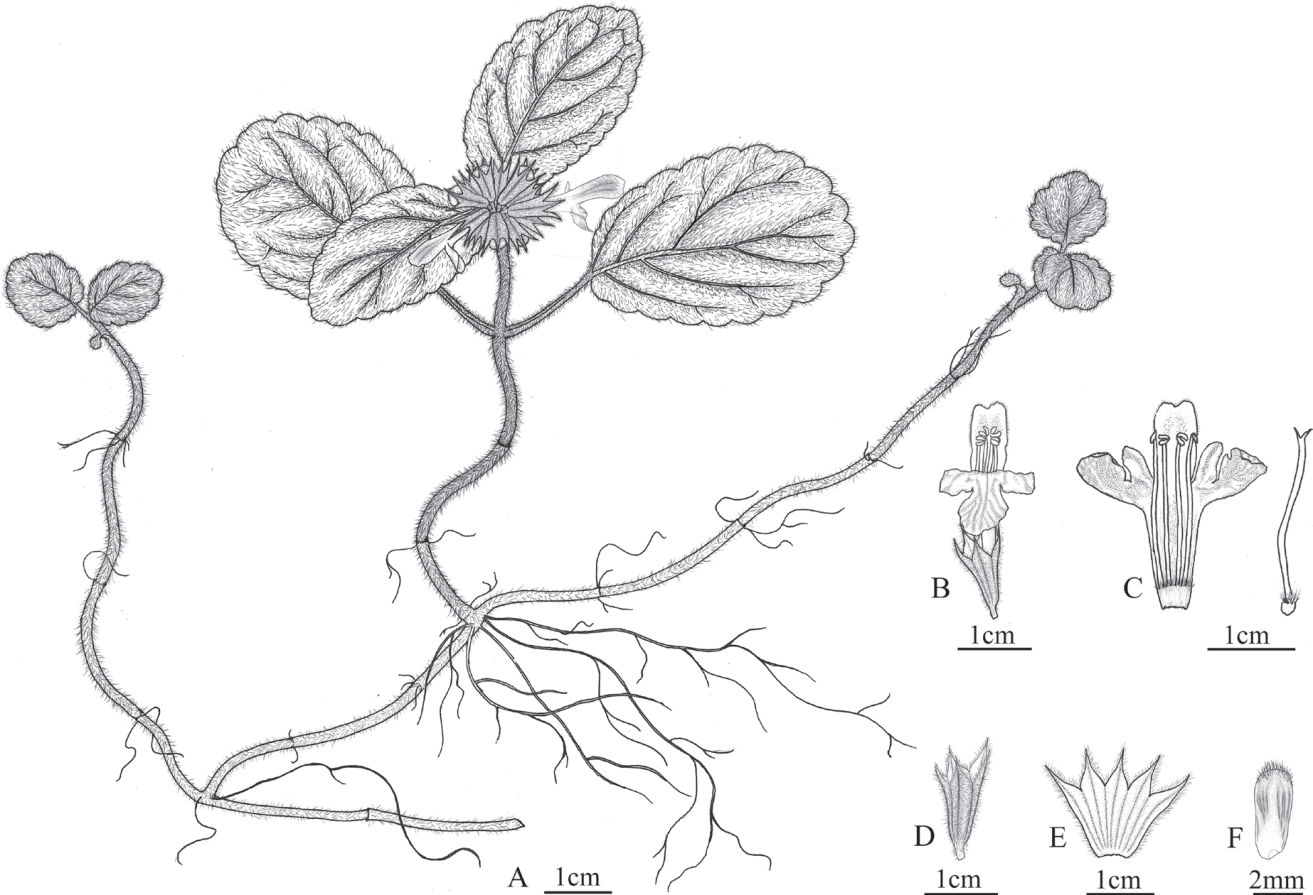
Line drawing of *Paraphlomis leigongshanensis*. **A.** Plant; **B.** Flower; **C.** Dissected corolla and pistil; **D.** Calyx; **E.** Dissected calyx; **F.** Nutlets. (Drawn by Hong-Mei Chen).

**Figure A2.**
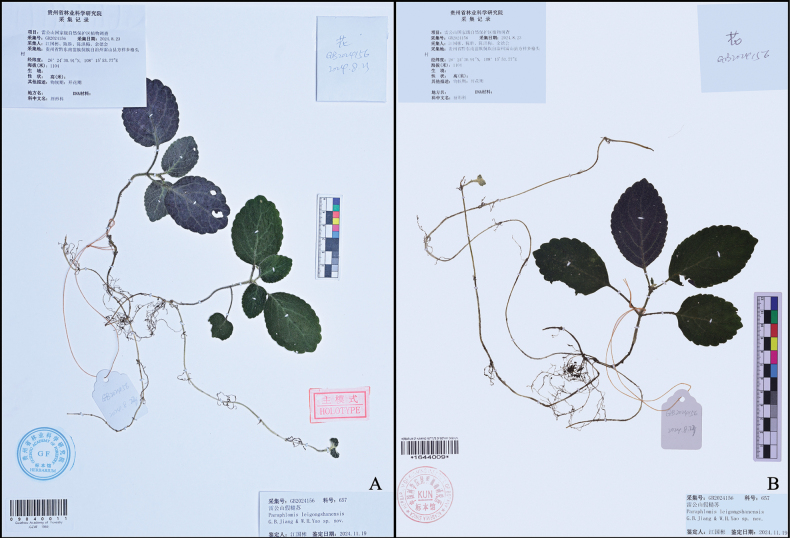
Holotype (**A**) and isotype (**B**) specimens of *Paraphlomis leigongshanensis*.

Figs 2, 3; Appendix 2: Figs A1, A2 雷公山假糙苏

##### Type.

China • Guizhou Province: Qiandongnan Miao and Dong Autonomous Prefecture, Leishan County, Leigong Mountain National Nature Reserve, 26°24'30.91"N, 108°15'53.77"E, alt. 1104 m, 23 Aug. 2024, *G.B. Jiang, S. Chen, H.M. Chen & D.H. Yu GB2024156* (holotype: GF 09040011!; isotypes: KUN 1644009!, HGAS 0128505!).

##### Diagnosis.

*Paraphlomis leigongshanensis* is morphologically most similar to *P. jiangyongensis* but can be distinguished by its calyx purplish red (vs. green), apex of calyx teeth lanceolate (vs. acute), corolla white with pink stripes (vs. pale yellow with red spots), and corolla tube 12–17 mm long (vs. 7.5–10 mm long).

##### Description.

Perennial herb, 5–10 cm tall, stoloniferous. ***Stems*** simple, prostrate to ascending, slightly 4-angled, densely villose. Leaves opposite, ovate to elliptical, papery, 2.5–8 × 2–5 cm; margin shallowly crenate, apex obtuse to subrounded, base broadly cuneate to rounded; adaxial surface green, abaxial purple, densely villose on both surfaces, particularly along veins, lateral veins 3–5-paired; petioles 0.5–3.5 cm long, densely villose. ***Verticillasters*** 2–10-flowered, sessile; bracteoles minute, early deciduous. ***Calyx*** purplish red, tubular-campanulate, 10–15 mm long, densely villose outside, glabrous inside; teeth 5, subequal, triangular, erect, 4–8 mm long, apex acuminate. ***Corolla*** white with pink stripes and spots, 2–3 cm long, densely pubescent outside; tube 1.2–1.7 cm long, slightly dilated at throat, pubescent annulate inside at 1/3 distance from base; bilabiate, upper lip pink, oblong, entire, erect, concave, 8–10 mm long, ca. 5 mm wide, lower lip pink, 3-lobed, medium lobe subcircular with pink stripes and spots, apex slightly emarginate, 6–7 mm wide, lateral lobes ovate, ca. 3.5 mm wide. ***Stamens*** 4, straight, included; filaments flat, pubescent at base; anther cells 2, parallel. ***Style*** included, glabrous, apex subequally 2-lobed, lobes subulate. ***Ovary*** densely pubescent and glandular at apex. ***Mericarps*** 4, dark brown, triquetrous-oblong, ca. 3.6 mm long, densely pubescent at apex.

##### Distribution, habitat and conservation status.

*Paraphlomis leigongshanensis* is currently known from only three populations in adjacent valleys approximately 100 m apart, within Leigong Mountain National Nature Reserve, Guizhou, China (Appendix 2: Fig. A1). The total population comprises about 200 mature individuals. The species occurs in the subtropical monsoon climate zone, with populations confined to the area near Getou Village, and is vulnerable to anthropogenic disturbances. Based on the IUCN Red List criteria B2(a, b(iii)) (extent of occurrence < 500 km^2^; < 5 locations; continuing habitat degradation), D (Estimated population size < 250 mature individuals), we proposed an Endangered (EN) assessment (IUCN 2024). Since similar adjacent habitats may harbor undiscovered populations, available evidence did not support Critically Endangered (CR) status.

##### Phenology.

Flowering was observed from May to August, and fruiting from August to October.

##### Etymology.

The specific epithet “*leigongshanensis*” is derived from the type locality of the new species, i.e., Leigong Mountain National Nature Reserve, Leishan County, Guizhou Province, China.

##### Chinese name (assigned here).

léi gōng shān jiă cāo sū (雷公山假糙苏). The name means a species of *Paraphlomis* only found in Leigong Mountain National Nature Reserve, Guizhou Province, China.

##### Additional specimens examined (paratypes).

China • Guizhou Province: Qiandongnan Miao and Dong Autonomous Prefecture, Leishan County, Leigong Mountain National Nature Reserve, 26°24'19.92"N, 108°15'19.20"E, alt. 1100 m, 1 Jul. 2025, *S. Chen & L. Hao GB2025015* (GF 09040012!, KUN 1644008!).

## Supplementary Material

XML Treatment for Paraphlomis leigongshanensis

## ﻿Additional information

### Use of AI

No use of AI was reported.

### Author ORCIDs

Wei-Hao Yao https://orcid.org/0009-0001-8231-0867

Guo-Bin Jiang https://orcid.org/0000-0002-4631-7906

De-Hui Yu https://orcid.org/0009-0008-4264-8995

Sheng Chen https://orcid.org/0000-0002-2727-6504

Hong-Mei Chen https://orcid.org/0009-0002-2368-7813

Li Dai https://orcid.org/0009-0004-3907-8582

Bai-Qiu He https://orcid.org/0009-0009-7491-3586

He Li https://orcid.org/0009-0006-0597-3209

### Data availability

The newly-obtained ITS and ETS sequences have been submitted to the NCBI (accession numbers: PV476761; PV478008). All of the other data that support the findings of this study are available in the main text.

## ﻿Appendix 1

**Table A1.** Sequence information for all samples used in present study. A “/” indicates a missing sequence. Herbarium abbreviations are listed after the vouchers. The accession numbers marked in bold represent sequences newly generated.

Taxon	Voucher	Country	ITS	ETS
Paraphlomis albida Hand.-Mazz. var. albida	A. Liu et al. LK0841 (CSFI)	Ningyuan, Hunan, China	MW602124	MW602091
Paraphlomis albida var. brevidens Hand.-Mazz.	Y.P. Chen EM312 (KUN)	Hezhou, Guangxi, China	MW602130	MW602098
*Paraphlomis albiflora* (Hemsl.) Hand.-Mazz.	C.M. Tan et al. 1806393 (JJF)	Jiujiang, Jiangxi, China	/	MW602101
*Paraphlomis baiwanensis* W.Y. Zhao, Y.P. Chen & Q. Fan 1	Y.S. Chen et al. QY20230302 (IBSC)	Qingyuan, Guangdong, China	PP897029	PP897950
*Paraphlomis baiwanensis* W.Y. Zhao, Y.P. Chen & Q. Fan 2	Q. Fan et al. 20251 (SYS)	Qingyuan, Guangdong, China	PP897030	PP897951
*Paraphlomis caloneura* K.J. Yan, Y.P. Chen & Y.F. Huang	W.H. Wu et al. LHT1841 (KUN)	Napo, Guangxi, China	OQ627454	OQ628080
*Paraphlomis chinensis* (Benth.) J.C. Yuan, Y.P. Chen & C.L. Xiang 1	Y. Yang OYY00316 (KUN)	Pingxiang, Jiangxi, China	MW602147	MW602117
*Paraphlomis chinensis* (Benth.) J.C. Yuan, Y.P. Chen & C.L. Xiang 2	Y. Yang OYY00131 (KUN)	Guilin, Guangxi, China	MW602148	MW602118
*Paraphlomis coronata* (Vaniot) Y.P. Chen & C.L. Xiang 1	E.D. Liu et al. 3043 (KUN)	Emeishan, Sichuan, China	MW602137	MW602107
*Paraphlomis coronata* (Vaniot) Y.P. Chen & C.L. Xiang 2	C.L. Xiang 358 (KUN)	Jiangkou, Guizhou, China	MW602123	MW602090
*Paraphlomis foliata* (Dunn) C.Y. Wu & H.W. Li	S.P. Chen s.n. (KUN)	Jiangle, Fujian, China	/	MW602097
*Paraphlomis gracilis* (Hemsl.) Kudô var. gracilis 1	A. Liu LK0931 (CSFI)	Changsha, Hunan, China	MW602134	MW602104
*Paraphlomis gracilis* (Hemsl.) Kudô var. *gracilis 2*	C.L. Xiang XCL1315 (KUN)	Chongqing, China	MW602141	MW602111
Paraphlomis gracilis var. lutienensis (Y.Z. Sun) C.Y. Wu	C.L. Xiang XCL881 (KUN)	Shibing, Guizhou, China	MW602131	MW602099
*Paraphlomis hirsutissima* C.Y. Wu & H.W. Li	F. Zhao & G. Chen XCL2115 (KUN)	Malipo, Yunnan, China	OQ627453	OQ628079
*Paraphlomis hispida* C.Y. Wu	X. Li LX200702 (GXF)	Napo, Guangxi, China	MW602132	MW602102
*Paraphlomis hsiwenii* Y.P. Chen & Xiong Li 1	W.H. Wu et al. DD426 (KUN)	Jingxi, Guangxi, China	OP605346	OP609841
*Paraphlomis hsiwenii* Y.P. Chen & Xiong Li 2	W.H. Wu et al. DD426 (KUN)	Jingxi, Guangxi, China	OP605347	OP609842
*Paraphlomis intermedia* C.Y. Wu & H.W. Li	X. Zhong et al. ZX16823 (CSH)	Suichang, Zhejiang, China	MW602135	MW602105
Paraphlomis javanica (Blume) Prain var. javanica 1	Y.P. Chen s.n. (KUN)	Kunming, Yunnan, China	MW602121	MW602088
Paraphlomis javanica (Blume) Prain var. javanica 2	L.B. Jia et al. JLB0029 (KUN)	Maguan, Yunnan, China	MW602143	MW602113
Paraphlomis javanica var. pteropoda D. Fang & K.J. Yan	X. Li 2020090501 (GXF)	Jingxi, Guangxi, China	MW602140	MW602110
*Paraphlomis jiangyongensis* X.L. Yu & A. Liu 1	A. Liu et al. LK1104 (CSFI)	Jiangyong, Hunan, China	MW602128	MW602095
*Paraphlomis jiangyongensis* X.L. Yu & A. Liu 2	A. Liu et al. LK1104 (CSFI)	Jiangyong, Hunan, China	MW602129	MW602096
*Paraphlomis kwangtungensis* C.Y. Wu & H.W. Li	Q. Fan et al. 19738 (SYS)	Qujiang, Guangdong, China	PP713070	PP706067
*Paraphlomis lanceolata* Hand.-Mazz. 1	C.Z. Huang s.n. (KUN)	Guidong, Hunan, China	MW602145	MW602115
*Paraphlomis lanceolata* Hand.-Mazz. 2	A. Liu et al. LK0825 (CSFI)	Ningyuan, Hunan, China	MW602146	MW602116
*Paraphlomis lancidentata* Y.Z. Sun	X. Zhong et al. ZX16824 (CSH)	Suichang, Zhejiang, China	MW602136	MW602106
***Paraphlomis leigongshanensis* G.B. Jiang & W.H. Yao**	**G.B. Jiang et al. GB2024156 (GF)**	**Leishan, Guizhou, China**	**PV476761**	**PV478008**
*Paraphlomis longicalyx* Y.P. Chen & C.L. Xiang	Y.P. Chen et al. EM583 (KUN)	Huanjiang, Guangxi, China	OK104771	OK104774
*Paraphlomis membranacea* C.Y. Wu & H.W. Li	M.S. Nuraliev 1057 (MW)	Thanh Son, Phu Tho, Vietnam	/	MW602100
*Paraphlomis montigena* (X.H. Guo & S.B. Zhou) J.C. Yuan, Y.P. Chen & C.L. Xiang	Y.C. Dai s.n. (KUN)	Hangzhou, Zhejiang, China	OM836064	OM884453
*Paraphlomis nana* Y.P. Chen, C. Xiong & C.L. Xiang 1	C. Xiong XC21097 (KUN)	Chengkou, Chongqing, China	OM836062	OM884451
*Paraphlomis nana* Y.P. Chen, C. Xiong & C.L. Xiang 2	C. Xiong & H.L. Zhou XC21126 (KUN)	Wushan, Chongqing, China	OM836063	OM884452
*Paraphlomis octopus* Q. Fan, Y.P. Chen & Ying Liu 1	Y.P. Chen & Y. Zhao EM1391 (KUN)	Huaiji, Guangdong, China	MW602126	MW602093
*Paraphlomis octopus* Q. Fan, Y.P. Chen & Ying Liu 2	Q. Fan et al. 19752 (SYS)	Fengkai, Guangdong, China	PP713071	PP706068
*Paraphlomis octopus* Q. Fan, Y.P. Chen & Ying Liu 3	Q. Fan et al. 19760 (SYS)	Pingle, Guangxi, China	PP713072	PP706069
*Paraphlomis pagantha* Dunn 1	L.X. Yuan et al. s.n. (KUN)	Qionghai, Hainan, China	OP605345	OP609840
*Paraphlomis pagantha* Dunn 2	X.Y. Jiang et al. HN001 (SYS)	Wenchang, Hainan, China	PP713073	PP706070
*Paraphlomis patentisetulosa* C.Y. Wu	C.L. Su et al. XY015 (KUN)	Xinyi, Guangdong, China	OQ627455	OQ628081
*Paraphlomis paucisetosa* C.Y. Wu 1	X.X. Zhu s.n. (KUN)	Malipo, Yunnan, China	MW602125	MW602092
*Paraphlomis paucisetosa* C.Y. Wu 2	X. Li LX200704 (GXF)	Napo, Guangxi, China	MW602133	MW602103
*Paraphlomis qingyuanensis* W.Y. Zhao, R.M. Wu & Q. Fan 1	Q. Fan et al. QYK-HH-1904 (SYS)	Yingde, Guangdong, China	PP897031	PP897952
*Paraphlomis qingyuanensis* W.Y. Zhao, R.M. Wu & Q. Fan 2	Q. Fan et al. 20255 (SYS)	Yingde, Guangdong, China	PP897032	PP897953
*Paraphlomis reflexa* C.Y. Wu & H.W. Li	Z.Z. Yang et al. s.n. (HIB)	Tongshan, Hubei, China	MW602122	MW602089
*Paraphlomis strictiflora* J.C. Yuan, B. Chen & C.L. Xiang	B. Chen et al. CB05956 (CSH)	Yinjiang, Guizhou, China	/	OP609839
*Paraphlomis subcoriacea* C. Y. Wu ex H. W. Li 1	Q. Fan et al. QYK-CJ-1911 (SYS)	Yangshan, Guangdong, China	PP897033	PP897954
*Paraphlomis subcoriacea* C. Y. Wu ex H. W. Li 2	Q. Fan et al. QYK-LB-1920 (SYS)	Yangshan, Guangdong, China	PP897034	PP897955
*Paraphlomis yangsoensis* (Y.Z. Sun) J.C. Yuan, Y.P. Chen & C.L. Xiang	L. Wu & W.B. Xu 10965 (IBK)	Yangshuo, Guangxi, China	MW602142	MW602112
*Paraphlomis yingdeensis* W.Y. Zhao, Y.Q. Li & Q. Fan	Q. Fan et al. 19013 (SYS)	Yingde, Guangdong, China	OP605348	OP609843
*Paraphlomis youyangensis* H. Jiang, R.B. Zhang & Tan Deng	R.B. Zhang et al. ZRB2531 (ZY)	Youyang, Guizhou, China	OR488121	OR487461
*Phlomis fruticosa* L.	Y. Tong s.n. (KUN)	Shanghai, China (cultivated)	MW602119	MW602086
Phlomoides dentosa var. glabrescens (Danguy) C.L. Xiang & H. Peng	Y.P. Chen EM360 (KUN)	Beijing, China (cultivated)	MW602120	MW602087

## ﻿Appendix 2

Supplementary figures provided in the present study.
